# Genome-wide association mapping and transcriptional analysis uncover genetic determinants of minor tocopherols in rice seeds

**DOI:** 10.1038/s41598-025-14473-3

**Published:** 2025-08-05

**Authors:** Sara Kazemzadeh, Naser Farrokhi, Asadollah Ahmadikhah, Pär K. Ingvarsson, Kourosh Tabar Heydar

**Affiliations:** 1 Department of Cell and Molecular Biology, Faculty of Life Sciences & Biotechnology, Shahid Beheshti University, Tehran, Iran; 2 Department of Plant Biology, Swedish University of Agricultural Sciences, Uppsala, Sweden; 3 Chemistry and Chemical Engineering Research Center of Iran, Tehran, Iran

**Keywords:** Tocopherol, *Oryza sativa*, GWAS, QTL, LncRNA, Protein kinase, Transcription factor, Transporter, Natural variation in plants, Plant breeding, Plant stress responses, Secondary metabolism, Quantitative trait

## Abstract

Despite the nutritional importance of tocopherols, current knowledge of the genetic architecture underlying the accumulation of minor tocopherols—gamma (γ) and delta (δ)—in rice (*Oryza sativa* L.) grains remains limited. In this study, we investigated the genetic basis of γ- and δ-tocopherol contents in rice using a genome-wide association study (GWAS) and post-GWAS analysis. Accordingly, 34,323 SNP markers were obtained from 179 genotypically diverse accessions of *O. sativa.* Minor tocopherol contents had a strong positive correlation (*r* = 0.76) with each other and varied greatly across the accessions: 0.015–1.74 and 0.005–0.81 (µg/g) for γ and δ, respectively. A total of 18 QTL on nine rice chromosomes were mapped. Eight transcription factor (TF) genes, five lncRNAs, and two transposons were found to be associated with the QTL. Moreover, three intracellular transport proteins were identified as associated genes with γ-tocopherol on chromosomes 1, 2, and 6. Protein kinases seem to have a substantiative function in defining the minor tocopherol contents, as they were associated with all 18 identified QTL. Haplotype analysis revealed that the QTL, namely *qDelt2.1*, *qGam2.1*, and *qGam6.1*, can maintain significant haplogroups. Comparative transcript analysis between high and low-content minor tocopherols demonstrated the possible involvement of *ERF71* (*Os06g0194000*), *TOR* (*Os05g0235300*) and *NAC70* (*Os02g0822400*) in defining the minor tocopherol contents in rice. Identified candidate genes in this study could be used in breeding programs to develop rice cultivars with high and beneficial levels of seed tocopherol contents.

**Supplementary Information:**

The online version contains supplementary material available at 10.1038/s41598-025-14473-3.

## Introduction

Tocopherols are a class of lipid-soluble, plastid-synthesized antioxidants present in all plant tissues, but most abundant in seeds where they are essential for protecting membrane lipids, during seed desiccation, storage, and germination^1^. Tocopherols consist of a polar chromanol head group, derived from homogentisic acid (HGA) and a lipophilic side chain derived from phytyl-diphosphate (PDP). The four tocopherols produced by plants (δ, β, γ and α) differ only in the numbers and positions of methyl groups on their chromanol head groups. Tocopherol biosynthesis initiates in plant cytoplasm, and the intermediate and the final steps of synthesis take place in plastids (**Supplementary Figure ****S1**) with necessary enzymes localized at the inner envelope or in the plastoglobules^2^. Although studies have focused more on α-tocopherol, recent studies have shown that the other isoforms of vitamin E such as gamma (γT) and delta-tocopherol (δT) also have useful properties^3^. Studies have shown that the antioxidant capacity of tocopherols is ranked as δ > γ > α > β at temperatures ranging from 80 to 120 °C, and α > γ > β > δ at temperatures between 20 and 60 °C^4^. The main forms of tocopherols in human diet are α- and γT, due to them being present in the highest content in food products. γT is the major form of vitamin E in many plant seeds, such as soybean^5^corn germ^6^rapeseed^7^flaxseed^8^sesame^9^ and the most abundant vitamin E in US diets. For example, in soybean oil γT accounts for more than 70%^10^. Like other vitamin E forms, γT is known to be an effective lipophilic antioxidant and capable of scavenging lipid peroxyl radicals. Studies show that γT has a strong anti-inflammatory activity and is related to the inhibition of carcinogenesis in humans^71–73^. On the other hand, γ-tocopherol, by forming 5-nitro-γT, can trap reactive nitrogen species and appears to show superior protection of mitochondrial function^11^.

δT is primarily found in castor oil and a lesser extent in wheat germ oil^12^. Identification of genes involved in biosynthetic pathways was traditionally reliant on classical biochemical methods such as protein purification and sequencing, which was a time-consuming and laborious process^74^. However, GWAS makes it possible to screen a very large number of accessions simultaneously to understand genetic contributions to metabolic diversity and their relevance to complex traits^13^. Linkage and association mapping studies of seed tocopherols have provided important insights into the genetic control of tocopherol synthesis that are generally consistent with transgenic studies. The association of seed longevity to γT and δT content in 185 diverse *AUS* rice varieties was reported by Lee et al. (2020)^14^. Wong et al. (2003)^15^ identified three QTL (Quantitative Trait Loci- genomic regions associated with variation in complex traits) on chromosomes 5 and 7, governing maize seed γT content. Furthermore, one QTL controlling seed δT content was identified and mapped on chromosome 5. Contrary to expectations, in maize seed, a non-photosynthetic organ, two chlorophyll biosynthetic enzymes were found to play a substantial role in explaining tocopherol diversity between genotypes^16^. Kassem et al. (2024) results were suggestive of the quantitative nature of δT and γT contents in maize seed^17^. Here, the possible regulatory mechanisms of δT and γT biosynthesis in rice were studied using GWAS and post-GWAS analyses in a natural rice population.

## Result

### Vitamin E contents in germplasm of rice grains

**Fig. 1:**
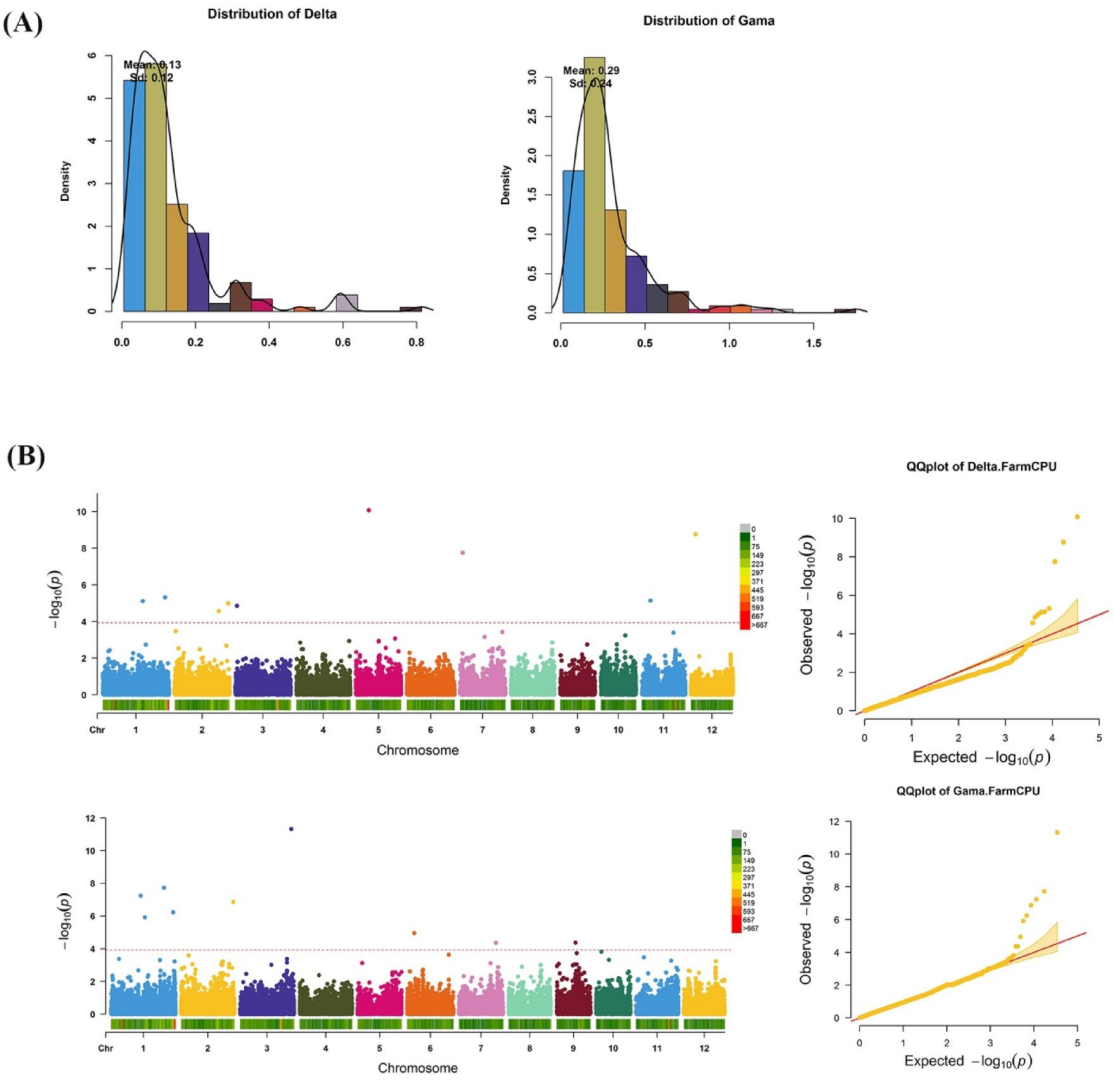
**(A)** Phenotypic distribution of δT and γT. **(B)** Manhattan and Q-Q plots for δT and γT. The FarmCPU model revealed 18 SNPs to be significantly associated (–log10(p) ≥ 4) with δT and γT content. The strongest association signal was detected for γT on Chr 3.

The total grain tocopherol content was quantified in 179 rice accessions via HPLC against standards of vitamin E isomers (δ, γ). The retention time was 10–15 min (**Supplementary Figure ****S2****)**. The rice accessions were classified into five specific categories, namely *Japonica*, *Indica*, *AUS*, *Aromatic*, and *Admix* according to Zhao et al., 2011^18^ (**Supplementary Table ****S1**). Gamma tocopherol (γT) content was significantly correlated with δT content (*r*^*2*^ = 0.77; **Supplementary Table ****S2**). The skewness and kurtosis of the association panel are shown in Fig. 1A.

A kinship matrix was used to summarize the distribution of pairwise relative relationship coefficients among the accessions in the association panel based on SNP information (**Supplementary Figure S3-A)**. A total of 34,323 SNPs distributed across 12 chromosomes were examined. The number of markers on each chromosome varied, with chromosome 9 having the fewest markers and chromosome 1 having the largest number **(Supplementary Figure S3-B).**

PCA using SNP data showed that three distinct subpopulations exist among the rice varieties in our core collection **(Supplementary Figure S3-C)**.

The γT content in the accessions varied widely from 0.015 to 1.740 (µg/g), with an average value of 0.280 (µg/g) and a coefficient of variation (C.V.) of 12%. δT content in the seeds of the 179 accessions varied from 0.005 to 0.810 (µg/g), with a mean value of 0.126 (µg/g) and a C.V of 17% **(Table **1**)**.

Estimates of broad-sense (H^2^b) and narrow-sense (H^2^n) heritabilities were 0.86 and 0.76 for γT and 0.87 and 0.79 for δT content, respectively. Estimated genotypic variance (V_G_) and additive variance (V_A_) accounted for γT were 29.75 and 26.64 and for δT were 30.71 and 27.88, respectively (**Table **1). Among the 179 rice accessions, the top five accessions for gamma isoform content were SungLiao2 (#g255), Sigadis (#g211), Pappaku (#g170), ParaibaChinesNova (#g171), Nortai (#g252) and for δT were CS-M3(#g10), Chibica (#g57), Sigadis (#g211), SungLiao2 (#g255), Pappaku (#g170).

### GWAS of vitamin E in rice grains

18 SNPs were identified as being significantly associated (–log_10_(*p*) ≥ 4) with δT and γT content, with nine SNPs associated with each trait **(Supplementary Table S3)**. Manhattan plot showed the distribution of the identified SNPs across the 12 rice chromosomes (Fig. 1B). Significantly associated SNPs are found on nine chromosomes, with only Chr. 4, 8 and 10 lacking any significant SNPs. A few QTL, such as *qGam2.1* and *qDelt2.1*, were co-located since they were significantly associated with both tocopherol components, likely due to pleiotropy (Table 2). The strongest association signal was detected for γT on Chr 3, with a SNP peak at locus id3016979 (–log_10_(*p*) = 11.3). Besides these QTL, the tocopherol *O*-methyltransferase (*OsVTE4*) and geranylgeranyl diphosphate synthase (*GGDP*) genes were also identified as being associated with δT content.

**Table 1:** Statistical and variation analysis of Tocopherol content in the tested rice population (*n* = 179).

Traits	Mean (µg/g)	Range (µg/g)	CV%	H^2^b	H^2^*n*	V_A_	V_G_	Skewness	Kurtosis	SE- Skewness	SE- Kurtosis
δT	0.126	0.005–0.810	17.0	0.87	0.79	0.019	0.021	2.7	12.3	0.182	0.361
γT	0.280	0.015–1.740	12.0	0.86	0.76	0.077	0.086	2.6	12.3		

Standard Error.

**Table 2:** Candidate genes identified via GWAS involved in vitamin E biosynthetic pathway.

Gene	SNP	Gene name	QTL name	Distance (kb)	LOP	Functional annotation	Reference
*Os02g0822100*	id2016014	OsAT1	*qGam2.1*	172	6.8	Increasing the grain size and weight	^37^
*Os01g0830000*	id1022369	ABCI5	*qGam1.1*	210 bp	7.7	ABC transporter family	-
*Os06g0198800*	id6003412	vesicle transport protein	*qGam6.1*	123	4.9	SNAP receptor activity	-
*Os05g0235300*	id5004299	TOR	*qDelt5.1*	181	10.07	As a developmental regulator in both plants and animals	^56^
*Os12g0162100*	id12001256	WNK9	*qDelt12.1*	105	8.7	Regulation of salt and drought response	^57^
*Os07g0131375*	wd7000267	OsPI4K2	*qDelt7.1*	69	7.75	Protein kinase activity	-
*Os07g0131500*	wd7000267	lectin-like receptor kinase		45	7.75	protein kinase activity	-
*Os07g0141100*	wd7000267	membrane-associated kinase-9		415	7.75	Serine/threonine-protein kinase, active	-
*Os07g0145400*	wd7000267	OsGHR1		605	7.75	Serine-threonine/tyrosine-protein kinase	-
*Os07g0133000*	wd7000267	lectin-like receptor kinase		32	7.75	Protein kinase superfamily	-
*Os07g0134200*	wd7000267	RLCK222		102	7.75	Seed development, drought toleranceroles, in stress defense	^58^
*Os07g0129800*	wd7000267	lectin-like receptor kinase		133	7.75	protein kinase activity	-
*Os07g0129900*	wd7000267	lectin-like receptor kinase		127	7.75	protein kinase activity	-
*Os07g0130400*	wd7000267	lectin-like receptor kinase		107	7.75	protein kinase activity	-
*Os07g0130800*	wd7000267	Protein kinase		97	7.75	protein kinase activity	-
*Os01g0931400*	id1025981	ROX1	*qDelt1.1*	89	5.3	thiamine diphosphokinase activity	^59^
*Os01g0927500*	id1025981	Ser Thr protein kinase		98	5.3	protein kinase activity	-
*Os01g0927900*	id1025981	OsAK2		74	5.3	aspartate kinase activity	^60^
*Os11g0212300*	id11002438	RLCK316	*qDelt11.1*	91	5.1	protein kinase activity	-
*Os11g0212900*	id11002438	OsWD40-187		118	5.1	protein kinase activity	-
*Os11g0208800*	id11002438	Receptor protein kinase		160	5.1	SR kinase	-
*Os11g0208900*	id11002438	Leucine rich repeat containing protein kinase		146	5.1	protein kinase activity	-
*Os11g0209700*	id11002438	OsPI4K2		85	5.1	kinase activit	^61^
*Os01g0648600*	id1015394	MAK2	*qDelt1.2*	24	5.1	drought tolerance	^62^
*Os02g0822500*	id2016014	Junctophilin type 2	*qDelt2.1*	195	4.9	phosphatidylinositol kinase activity	-
*Os02g0822900*	id2016014	RLCK91		222	4.9	salt tolerance	-
*Os02g0819600*	id2016014	RLCK88		76	4.9	bacterial blight disease resistance	^63^
*Os02g0819900*	id2016014	RLCK89		88	4.9	Receptor-like Cytoplasmic Kinase 89	-
*Os02g0821400*	id2016014	RLCK90		147	4.9	bacterial blight disease resistance, seed development trait	-
*Os02g0815900*	id2016014	Protein kinase		165	4.9	protein kinase activity	-
*Os03g0127700*	id3000735	LRR receptor-like kinase		315	4.8	kinase activity	-
*Os03g0118400*	id3000735	CDC2-1	*qDelt3.1*	245	4.8	CELL DIVISION CONTROL PROTEIN 2 HOMOLOG 1	-
*Os03g0122000*	id3000735	Ser Thr protein kinase		28	4.8	kinase activity	-
*Os03g0124200*	id3000735	CRRLK1L6		162	4.8	CATHARANTHUS ROSEUS RECEPTOR-LIKE KINASE1-LIKE KINASE 6	-
*Os03g0125400*	id3000735	D-erythro-sphingosine kinase		205	4.8	kinase activity	-
*Os03g0125600*	id3000735	Ser Thr protein kinase		215	4.8	protein kinase activity	-
*Os02g0700600*	id2012639	CDKF	*qDelt2.2*	75	4.5	Serine/threonine-protein kinase	-
*Os02g0694900*	id2012639	Protein kinase		344	4.5	protein kinase activity	-
*Os02g0698000*	id2012639	OsPrk		197	4.5	Phosphoribulokinase/uridine kinase	-
*Os03g0821900*	id3016979	RLCK118	*qGam3.1*	95	11.3	bacterial blight disease resistance, brassinosteroid sensitivity	^64^
*Os03g0825300*	id3016979	RLCK119		65	11.3	salt tolerance, cold tolerance	-
*Os03g0825800*	id3016979	RLCK120		87	11.3	seed development trait	-
*Os03g0828800*	id3016979	SDRLK60		210	11.3	protein kinase activity	-
*Os02g0815900*	id2016014	Protein kinase	*qGam2.1*	165	6.8	Serine/threonine-protein kinase	-
*Os02g0819600*	id2016014	RLCK88		76	6.8	bacterial blight disease resistance	^63^
*Os02g0819900*	id2016014	RLCK89		88	6.8	bacterial blight disease	-
*Os02g0821400*	id2016014	RLCK90		147	6.8	bacterial blight disease resistance, seed development	-
*Os02g0822900*	id2016014	RLCK91		222	6.8	salt tolerance	-
*Os01g0832300*	id1022369	CDPK3	*qGam1.1*	117	7.7	protein kinase activity	-
*Os01g0832900*	id1022369	OsSTK1		149	7.7	Serine/threonine-protein kinase	-
*Os01g0541900*	wd1001380	PP2C4	*qGam1.2*	115	7.2	Serine/threonine-protein kinase	-
*Os01g0581400*	id1012891	RLCK39	*qGam1.4*	181	5.9	seed development trait	-
*Os01g0587400*	id1012891	SDRLK49		172	5.9	submergence tolerance	^62^
*Os01g0588500*	id1012891	RLCK40		200	5.9	osmotic response sensitivity, blast disease, heat tolerance seed development trait	-
*Os06g0198900*	id6003412	RLCK202	*qGam6.1*	126	4.9	Receptor-like Cytoplasmic Kinase 202	-
*Os07g0602700*	id7004575	Protein kinase	*qGam7.1*	132	4.3	protein kinase activity	-
*Os03g0822100*	id3016979	Similar to Transposase	*qGam3.1*	76	11.3	DNA binding	-
*Os01g0540400*	wd1001380	MuDR	*qGam1.2*	1	7.2	-	-
*Os03g0820400*	id3016979	ZFP15	*qGam3.1*	174	11.3	related to stress response	^65^
*Os03g0820300*	id3016979	Zinc Finger 182		177	11.3	drought-resistant	^66^
*Os03g0818700*	id3016979	MYB		252	11.3	-	-
*Os03g0818800*	id3016979	EREBP33		244	11.3	Salt tolerance, grain size	^67^
*Os02g0822400*	id2016014	NAC70	*qGam2.1*,* qDelt2.1*	189	6.8	Drought tolerance, salt tolerance	^68^
*Os01g0584900*	id1012891	WRKY77	*qGam1.4*	4	5.9	Salt tolerance, drought tolerance, cold tolerance, bacterial blight disease resistance	^69^
*Os06g0194000*	id6003412	ERF71	*qGam6.1*	160	4.9	Jasmonic acid sensitivity, drought tolerance	^70^
*Os02g0698800*	id2012639	WRKY66	*qDelt2.2*	187	4.5	Bacterial blight disease resistance, heat tolerance	-
*Os02g0821301*	id2016014	URS00008E75A9_39947	*qGam2.1*,* qDelt2.1*	147	6.8	Non-protein coding	-
*Os07g0604466*	id7004575	URS00008F1D21_39947	*qGam7.1*	0	4.3	Non-protein coding	-
*Os12g0158500*	id12001256	URS00008FD455_39947	*qDelt12.1*	98	8.7	Non-protein coding	-
*Os06g0192676*	id6003412	URS00008F2D91_39947	*qGam6.1*	220	4.9	Non-protein coding	-
*Os07g0604900*	id7004575	URS0000180188_39947	*qGam7.1*	19	4.3	Non-protein coding	-

**LOP**: -Log_10_ (P).

**The distance**: Physical distance between each gene and the peak SNP within the associated QTL region.

### SNP annotation

Functional annotations of the identified SNPs using SnpEff revealed that all associated SNPs were located outside of the coding sequences, with the exception of id1022369 that was located in the promoter region *ABCI5*. The *cis*-acting element of the *ABCI5* promoter was WAACCA and *MYB1AT* is the *trans*-acting factor.

### Candidate genes identification

Linkage disequilibrium analysis revealed that LD dropped to half of its maximum value at a distance of 300–1300 kb across the 12 rice chromosomes (**Supplementary Figure S4**). The flanking regions (SNP position ± average distance of LD decay) for the 18 QTL were checked for the presence of the putative associated genes. Various regulatory elements (TFs, LncRNA and transposons) and functional genes with cellular transport and signaling roles were found in the close vicinity of the mapped QTL (**Supplementary Table S4)**. Promoter analysis of the tocopherol biosynthetic pathway genes (*vte1*,* vte2*,* vte3*,* vte5*,* vte6*,* hppd*,* tat and ggpps*) was carried out using 1500 nucleotide^75,76^ windows upstream of the start codon for each gene to determine if corresponding *cis*-elements are present. The genes *ZFP15*, *ZFP182*, *MYB*, *EREBP33*, *NAC70*, *WRKY77*, *ERF71*, *WRKY66* have at least one corresponding binding site in the promoter region of the different genes in the tocopherol biosynthesis pathway (**Supplementary Table S5**) and introduced as candidate TFs with probable roles in controlling tocopherol content (Table 2).

Two transposons (*Os03g0822100* and *Os01g0540400*) belonge to *qGam3.1* and *qGam1.2* QTL, on chromosomes 3 and 1 were found to be associated with the content of γT. The transporter genes *OsAT1*, *ABCI5* and *Os06g0198800*, belonge to *qGam2.1*, *qGam1.1*, *qGam6.1* QTL, located on chromosomes 2, 1 and 6, were also found to be also associated to γT content. These genes belong to the SLC26, SNARE and ABC transporter families **(**Table 2**)**. Protein kinases were also associated with 16 QTL likely to act as key signaling factors **(**Table 2**)**.

### miRNA-lncRNA-mRNA network

We selected 18 QTL and 72 non-protein coding transcripts (36 each for δT and γT) that were found to be located in flanking regions of SNP markers. Following a thorough search in PLncDB V2.0, 57 primary lncRNAs (28 for δT and 29 for γT) were retrieved (**Supplementary Table S6**). To evaluate the potential role of these lncRNAs as targets of miRNA molecules, the primary sequences of the lncRNA were aligned to identified miRNA molecules that have been registered for rice in miRBase. A total of 54 specific miRNAs (27 for δT and 27 for γT) were identified in this manner **(Supplementary Table S7)**.

Furthermore, the target mRNAs for miRNAs were identified by psRNATarget by giving miRNA sequences as input, searching against mRNA sequences of the 13 main vitamin E biosynthetic genes^21^ and 20 transcription factors. Five mRNAs [(*WRKY39*: *Os02g0265200*), (*NAC70*, *Os02g0822400*), (*MYB*-*like*,* Os03g0818700*), (*EREBP33*, Os03g0818800), (*OsHGGT*, Os06g0646900)] are acting as targets for six miRNAs (*osa-miR5075*, *osa-miR1436*, *osa-miR2919*, *osa-miR172a*, *osa-miR172b*,* osa-miR2102*-*5p*) **(Supplementary Table S8).** lncTAR was used to identify the mRNA targets of lncRNAs, and target mRNAs were identified for 12 lncRNAs (**Supplementary Table S9**).

Co-expression analysis for TFs was performed in the RiceFREND database, with the results summarized in **Supplementary Table S10**, which lists the TFs along with their co-expressed genes, highlighting potential regulatory networks and functional associations.

**Fig. 2:**
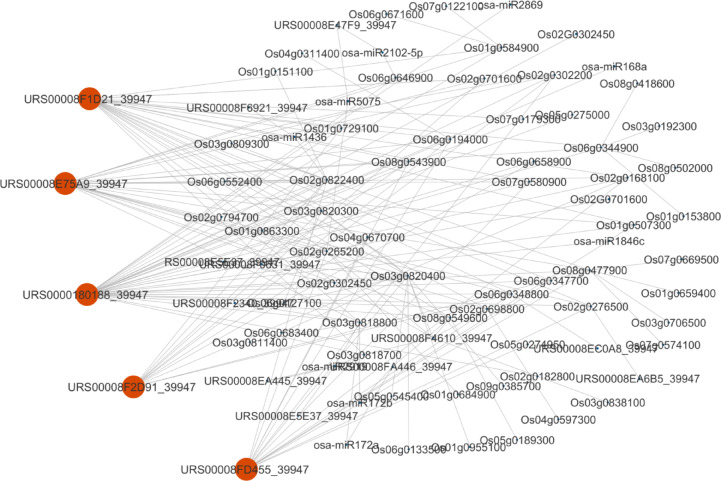
The network of lncRNA, miRNA, and mRNA interactions. Red circles are *URS00008E75A9_39947*,* URS00008F1D21_39947*,* URS00008FD455_39947*,* URS00008F2D91_39947* and *URS0000180188_39947* with the highest degree of connectivity as the candidate genes (created using Cytoscape _v3.9.1).

An interaction network was constructed for lncRNA, miRNA and mRNA using Cytoscape _v3.9.1 **(**Fig. 2**).** Five key lncRNAs, namely *Os02g0821301* (*URS00008E75A9_39947*, associated with γ and δ; 147,424 bp), *Os07g0604466* (*URS00008F1D21_39947*, γ; 0 bp), *Os012g0158500* (*URS00008FD455_39947*, δ; 98,853 bp), *Os06g0192676* (*URS00008F2D91_39947* γ; 220,703 bp), and *Os07g0604900* (*URS0000180188_39947* γ; 19,084 bp) were identified **(**Table 2**)**.

### Epistasis among QTLs of γT and δT

**Fig. 3:**
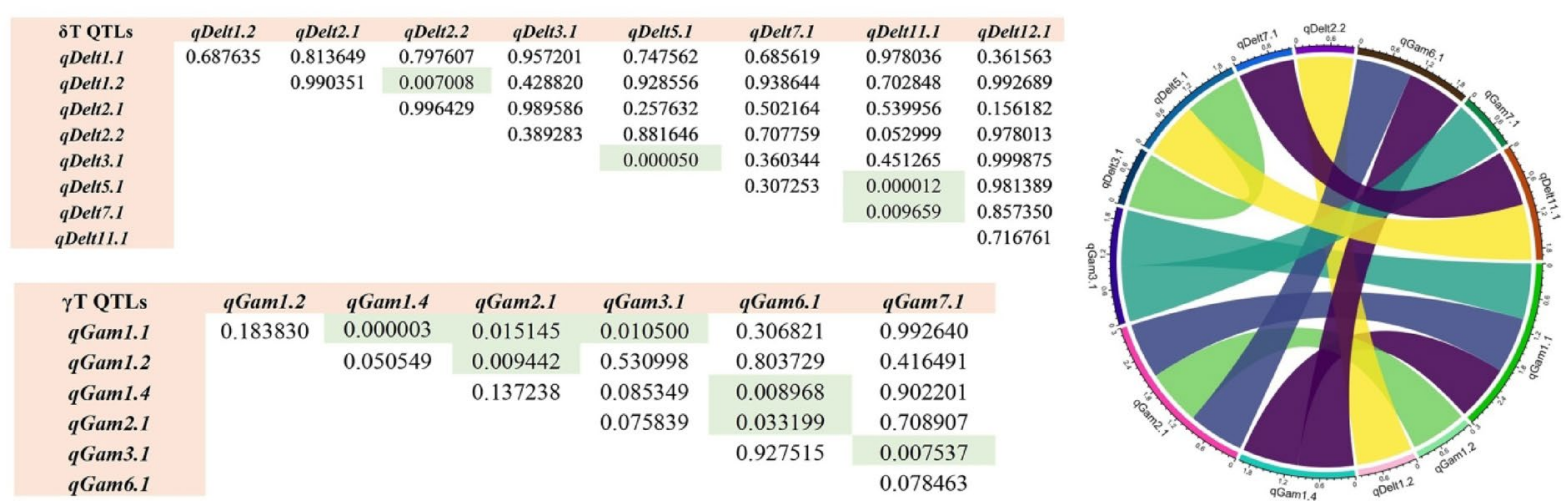
Evidence of epistatic effects between different δT and γT QTL. Interactions ≤ 0.05 are significant and are highlighted with a grey box. Circos plot of epistasis interaction between QTL for δT and γT detected from the GWAS result. QTL that had significant epistatic associations are located around the circle. The Circlize package was used to visualize the significant epistatic interactions.

A total of 11 QTLs (SNP position ± average LD decay) showed significant epistatic effects (Fig. 3), involving several QTL pairs. These included *qGam1.1* (*ABCI5*, *CDPK3*, *OsSTK1*) with *qGam1.4* (*RLCK39*, *SDRLK49*, *RLCK40*, *WRKY77*); *qGam1.1* with *qGam2.1* (OsAT1, *RLCK88*–*91*, *Os02g0821301*); and *qGam1.1* with *qGam3.1* (*RLCK118*–*120*, *SDRLK60*, *ZFP15*, *ZincFinger182*, *MYB*, *EREBP33*).

Additional interactions were observed between *qGam1*.*2* (*PP2C4*, *MuDR*) and *qGam2*.*1* (*OsAT1*); *qGam1*.*4* and *qGam6*.*1* (*RLCK202*, *ERF71*, *Os06g0192676*); *qGam2*.*1* and *qGam6*.*1;* as well as *qGam3*.*1* and *qGam7*.*1* (*Os07g0604466*, *Os07g0604900*). These epistatic pairs were associated with variation in γ-tocopherol content (*p* ≤ *0.05*).

For δ-tocopherol (*p ≤ 0.05*), epistatic interactions were detected between *qDelt1*.*2* (*MAK2*) and *qDelt2*.2 (*CDKF*, *OsPrk*); *qDelt3*.*1* (*CDC2*-*1*, *CRRLK1L6*, *D*-*erythro*-*sphingosine kinase*) and *qDelt5*.*1* (*TOR*); *qDelt5*.*1* and *qDelt11*.*1* (*RLCK316*, *OsWD40*−187, *OsPI4K2*); and finally *qDelt7*.1 (*OsPI4K2*, *RLCK222*) with *qDelt11*.*1* (Fig. 3). There are several genes in each QTL and different genes can have different epistatic effects, unfortunately, we could not determine the type of effect. The results presented here indicate that epistatic interaction plays an important role in controlling the expression of complex traits and marker-assisted selection in plant breeding programs should consider including epistatic effects to increase selection accuracy.

### Haplotype analysis

**Fig. 4:**
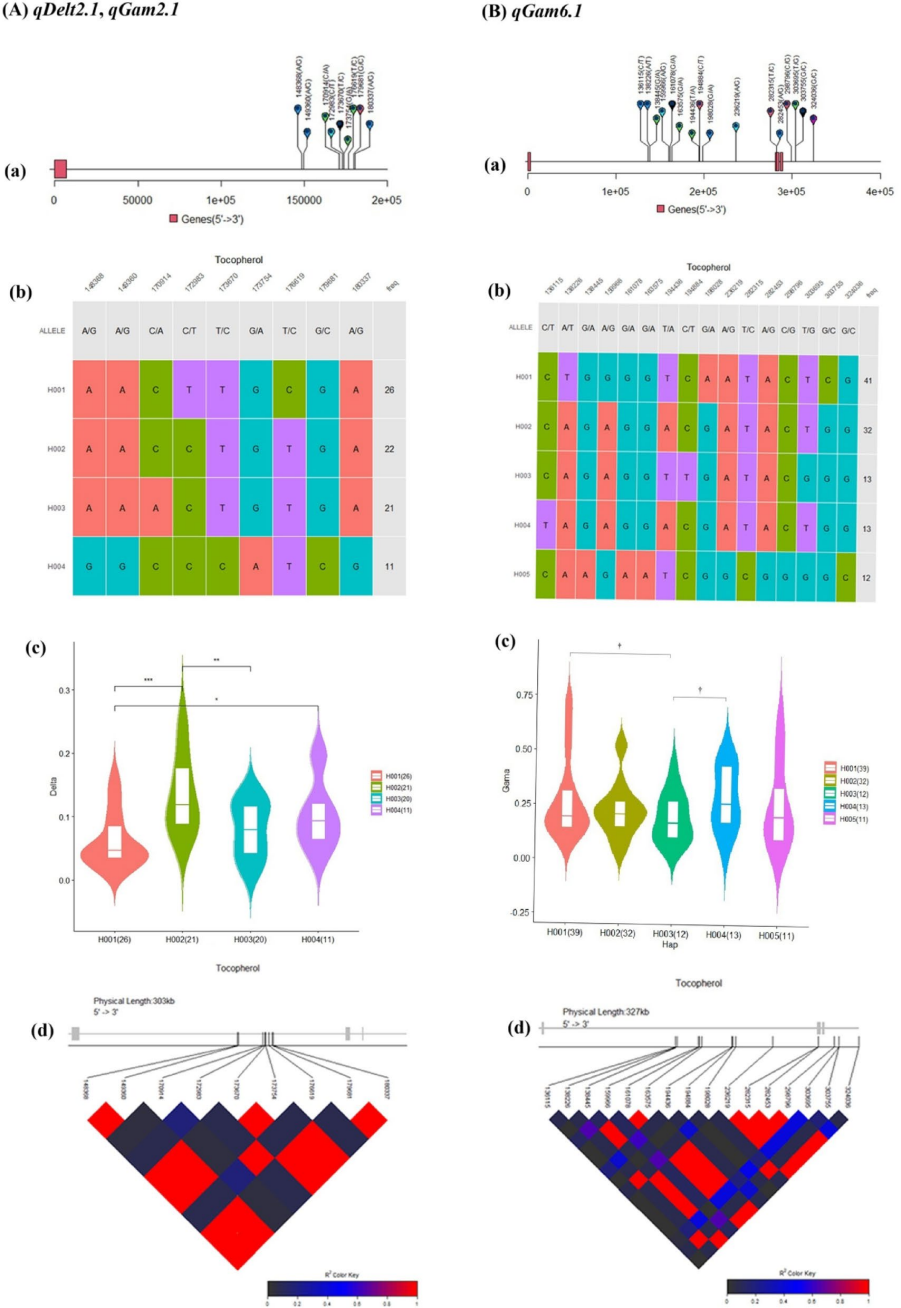
Haplotype analyses for different QTL related to δT and γT. **(A)** *qDelt2.1*,* qGam2.1*, **(B)** *qGam6.1* (**a**) Visualization of SNP position above gene model, the black line represents the genome and rectangles represent exons; (**b**) Haplotype classification, each row represents a haplotype, colored columns represent loci, and the last column shows the frequency of each haplotype; (**c**) Phenotype comparisons among accessions possessing different haplotypes; (**d**) LD-block visualization of SNP sites in the locus. * Indicates: *p* < 0.05, ** indicates: *p* < 0.01, *** indicates: *p* < 0.001, † significant at *p* ≤ 0. 1. The red color indicates perfect LD, and the black color indicates no LD.

Haplotype analysis was conducted using all the QTL, while only statistically significant QTL are being reported here. Haplotype analysis of QTL *qDelt2.1*,* qGam2.1* which were associated with δT and γT on chromosome 2 formed a haplotype block with nine SNP markers. The SNP markers resulted in four haplogroups in our association panel (Fig. 4A-a, b). Variation in these haplotype alleles lead to significant differences (*p* ≤ 0.05) between the H001/H004, H002/H003 (*p* ≤ 0.01) and H001/H002 (*p* ≤ 0.001) haplotypes for both γT and δT. The median δT and γT values in the violin plot of the haplogroups were 0.06 (H001), 0.13 (H002), 0.08 (H003), 0.09 (H004) µg/g, respectively. The highest amount of tocopherol was observed for haplotype H002 (Fig. 4A-c). H001 had the highest frequency (32.5%) in our association mapping panel and was mostly represented by *Japonica* (*TEJ*) varieties, based on the haplotype network (**Supplementary Figure S5**).

Haplotype analysis of *qGam6.1* resulted in the formation of five haplogroups among the 179 rice accessions, consisting of 16 SNPs in LD on chromosome 6. The highest amount of tocopherol was observed for haplotype H004. The median of H001 and H003 were 0.19 and 0.16 µg/g, respectively, showing significant differences (*p* ≤ 0.1). Also, H003 and H004 showed significant differences (*p* ≤ 0.1). The highest haplotype frequency was observed for H001 (41%), which was represented by *Japonica* (*TEJ* and TRJ), *Admix* and *Indica* varieties (Fig. 4B-a-d, **Supplementary Figure S5)**.

### PPI network analysis

**Fig. 5:**
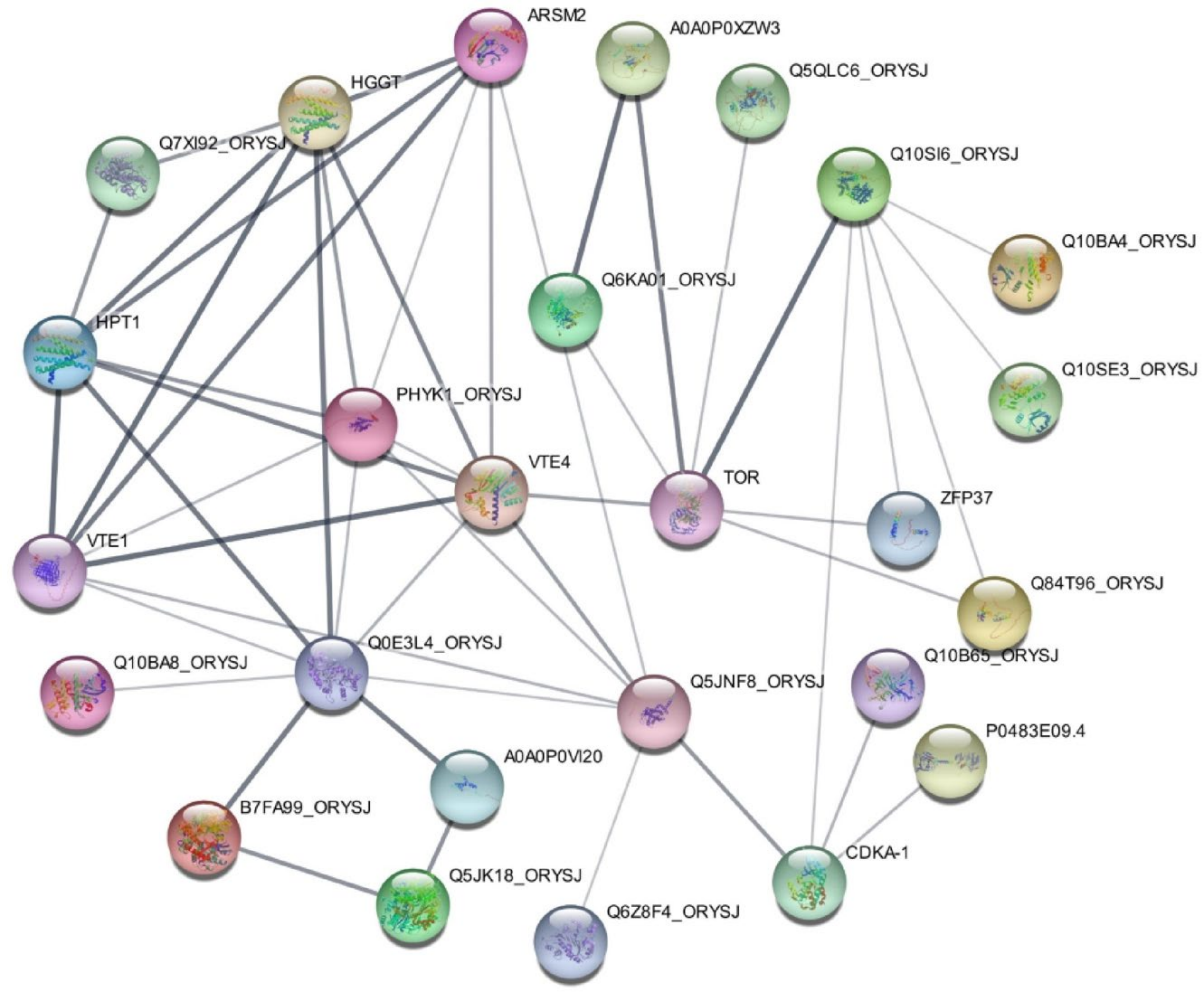
Protein association networks between candidate genes and main genes involved in tocopherol biosynthesis. The results showed that *TOR* is co-expressed with *VTE4* and *RLCK119* is co-expressed with HPPD. *CDKA-1* and *OsPrk* are co-expressed with *VTE6*. Also, *OsAK2* is co-expressed with *TAT-1* and *TAT-2.*

Protein-protein interaction (PPI) networks analysis of candidate genes and the main biosynthetic genes involved in tocopherol biosynthesis revealed that *TOR* (*Os05g0235300*) is co-expressed with *VTE4* in the first hierarchy. Based on this result, the *TOR* gene (member of signaling category) was selected as a candidate for real-time qRT-PCR analysis (Fig. 5).

### RNA-Seq analysis of the candidate genes

**Fig. 6:**
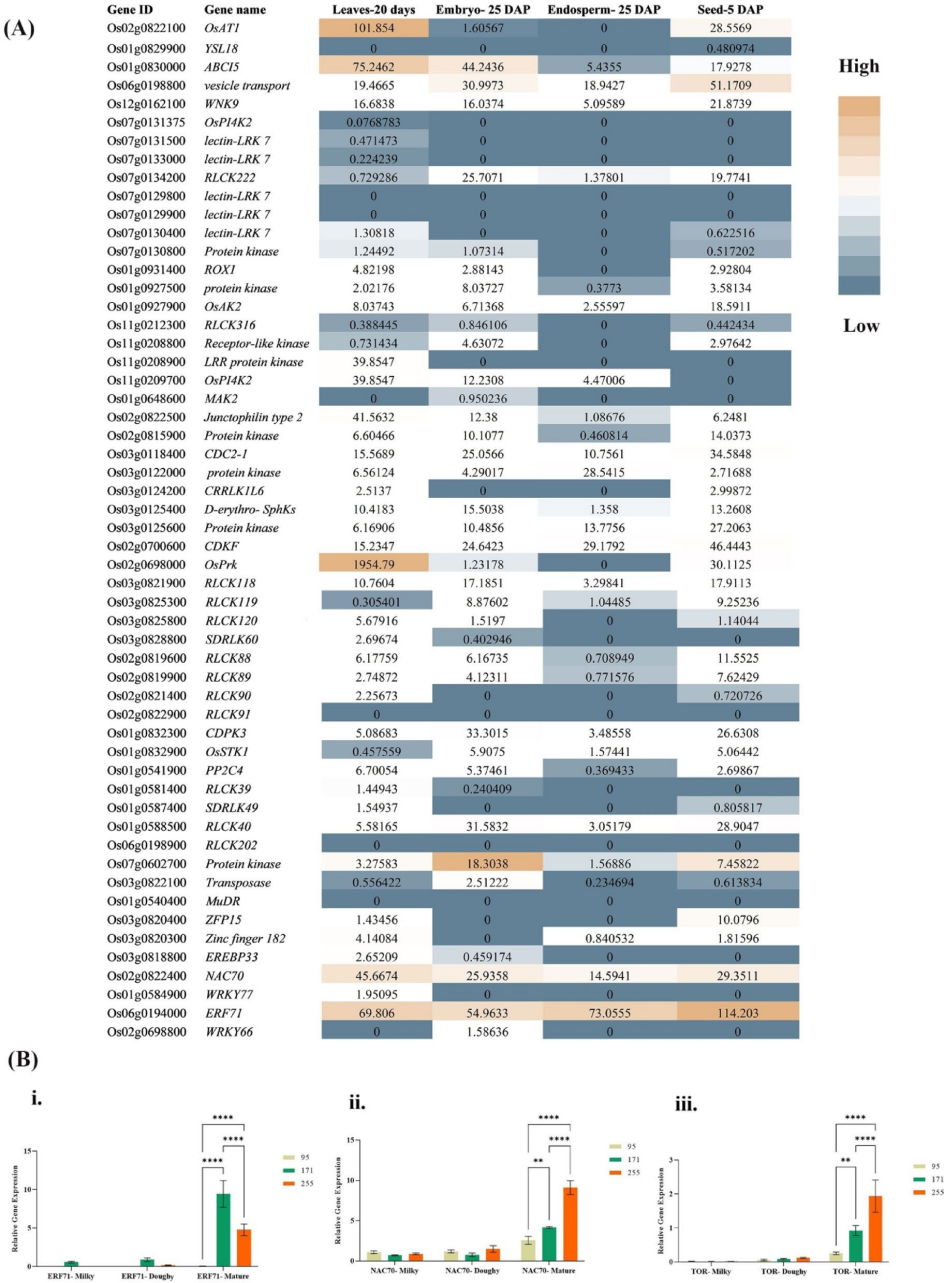
**(A)** Heatmap of candidate gene expression analysis by RNA-seq data obtained from RGAP database for leaves (20-day-old), embryo- 25 DAP, endosperm- 25 DAP and seed 5 DAP. Dark orange boxes indicate high transcript levels and blue boxes indicate low transcript levels. (**B)** Expression analysis of **(i)** *ERF71* (*Os06g0194000*), **(ii)** *NAC70* (*Os02g0822400*) and **(iii)** *TOR* (*Os05g0235300*) by real-time qRT-PCR. Amplification of cDNA from three developmental stages of seeds, i.e., milky, doughy, and maturity in three rice genotypes (#g95, #g255, #g171) content. A housekeeping gene, *OsActin*, was used as the control and the expression data was compared to the milky stage. Data were analyzed using a two-way ANOVA, followed by Tukey’s multiple comparison test with a 95% confidence interval. Significantly different comparisons between #g95 vs. #g255 and #g171 at stages of seed growth are displayed on graphs using asterisks (****, *P* < 0.0001, **, *P* < 0.01). No asterisk indicates that the difference is not significant. Error bars represent mean ± SD (*n* = 3 biological replicates).

The results of RNA-seq data from diverse rice tissues showed that among the transporter genes, *Os06g0198800* showed high expression at the seed-5 DAP (days after pollination) stage. *OsAT1* was weakly expressed in the seed-5 DAP stage but showed the highest expression in the leaves-20 days stage. The highest expression of the *ABCI5* transporter was observed in the leaves-20 days stage. Among the signaling genes, the *Os07g0602700* protein kinase showed slight expression in seeds and had its highest expression in the embryo-25 DAP stage. The highest expression observed for the signaling genes belonged to *OsPrk* in leaves. Other effective genes in signaling were expressed in small amounts in different tissues. No significant expression was seen among transposons in this study. Among TFs, *ERF71* and *NAC70* showed expression in all tissues. The highest expression of *ERF71* was in seed. Also, *ZFP15* was slightly expressed in the seed. *ERF71* and *NAC70* were considered the regulatory factors and were selected as the candidates for validation using real-time qRT-PCR (Fig. 6A).

### Quantitative real-time PCR (qRT-PCR)

The qRT-PCR results showed that the expression levels of all three selected genes *ERF71* (*Os06g0194000*), *NAC70* (*Os02g0822400*) and *TOR* (*Os05g0235300*) were significantly higher in genotypes #g171 and #g255 (high tocopherol) than #g95 (low tocopherol) at mature stages (Fig. 6B **i**,** ii**,** iii**). A two-way ANOVA analysis showed that at the mature stage, there were significant differences in the expression of the *ERF71* gene between #g95 and #g171 (*p* < 0.0001), #g95 and #g255 (*p* < 0.0001) and #g171 and #g255 (*p* < 0.001). Similarly, for the *NAC70* gene, there were significant differences in expression between genotypes #g95 and #g171 (*p* < 0.01), #g95 and #g255 (*p* < 0.0001) and #g171 and #g255 (*p* < 0.0001). The analysis also revealed significant differences in the expression of the *TOR* signaling gene between #g95 and #g171 (*p* < 0.01), #g95 and #g255 (*p* < 0.0001) and #g171 and #g255 (*p* < 0.0001). However, none of these three genes showed significant differences in the doughy and milky stages. These results suggested that *ERF71*, *TOR* and *NAC70* may play a crucial role in tocopherol biosynthesis in rice seed.

### Gene enrichment analysis

**Fig. 7:**
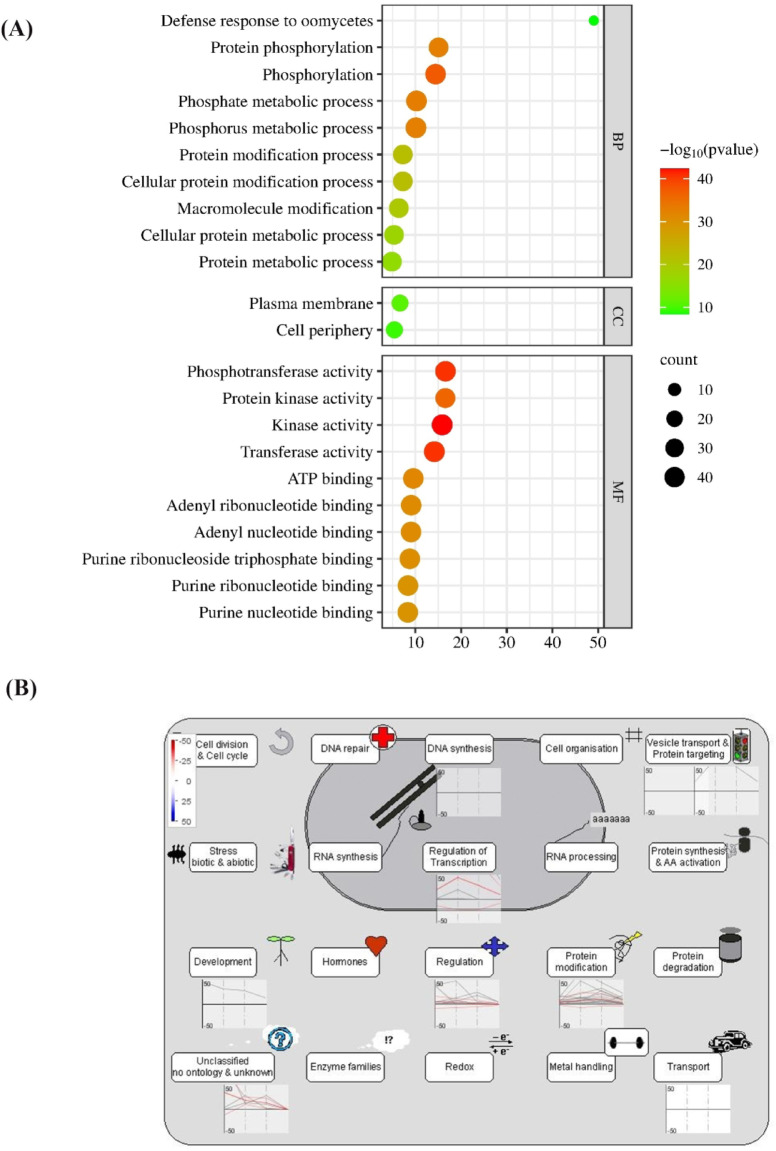
**(A) (**GO analysis revealed that candidate genes were significantly enriched in biological processes, cellular components, and molecular functions. GO, Gene Ontology (www.bioinformatics.com.cn/srplot). (**B)** MapMan analysis. Cell function overview associated with candidate gene. The studied genes are mainly effective in the regulation, protein modification, transport, and regulation of transcription, which are the precursors of tocopherol biosynthesis. For each functional group, the genes that have an increase and decrease are shown as a series of blue and red lines. MapMan software (ver. 3.6.0RC1) was used to map transcriptomic data. Osa_RAPDB_mapping files and were downloaded from the Map-Man store server (http://mapman.gabipd.org/).

Gene Ontology (GO) analysis revealed that in the biological process (BP) category, the differentially expressed genes (DEGs) were mainly enriched for involvement in the protein metabolic process, cellular protein metabolic process, macromolecule modification, protein modification process, cellular protein modification process, phosphorus metabolic process, phosphorylation, defense response to oomycetes (Fig. 7A). The GO analysis further unveiled that for cellular components (CC), the candidate genes (Table 2) exhibited prominent enrichment in various CC terms. This included plasma membrane and cell periphery (Fig. 7A). The molecular functions (MF) of the candidate genes included phosphotransferase activity, protein kinase activity, transferase activity, ATP binding, purine ribonucleotide binding and adenyl nucleotide binding (Fig. 7A).

An overview of candidate genes expression provided in Fig. 7B, shows that they are sorted into 8 BINs or subBINs that reflect major cellular or functional processes. A high proportion of genes were called present for signaling (BinCode: 30), protein-post translation modification (BinCode: 29.4), RNA-regulatory of transcription (BinCode: 27.3), transport (BinCode: 34), development (BinCode: 33), protein targeting (BinCode:29.3), DNA synthesis (BinCode: 28.1.1.4) and BIN 35.2 (unknown) that were not placed in any group. Marked changes in the expression pattern were found in all of these categories.

## Discussion

Rice is an important crop for food security and has also been used as a model crop in many genetic studies^22^. Tocopherols are supplied in various amounts in a diet, mainly from vegetable oils, oilseeds, and nuts. The amount of α-tocopherol in rice seed is higher than the other isoforms while γT and δT are the minor components of vitamin E content in rice seed. Here, we used GWAS to identify QTL and candidate genes involved in regulating the signaling and transport of γT and δT in a natural rice population.

We observed wide variation in γT and δT contents among the seeds from diverse rice accessions (*Indica*,* Japonica*,* AUS*,* Admix*, and *Aromatic*). Both γT and δT had high heritabilities, meaning that these traits are subject to little variation from environmental factors and genetic analyses can hence be used to dissect the genetics underlying the diversity of the traits seen among accession in our rice population (**Table 1**). GWAS is an effective method to identify QTL linked to traits of interest and to explore the tocopherol diversity in the rice grain we employed a panel of 179 diverse rice genotypes where we scored minor tocopherols via HPLC equipped with a fluorescent detector. The association analysis used 34,323 SNPs and the FarmCPU model identified 18 SNPs that were considered significantly associated with the two traits (-Log_10_(*P*) ≥ 4). Generally, the amounts of tocopherols are higher in seed bran, but we were not able to separate the bran from seeds and therefore, our results may differ slightly from an earlier study by Wang et al. (2015)^75^.

Epistasis, or locus-locus interactions^23^is known to play an important role in phenotypic variation and has received much attention in recent years. As a major factor in molecular evolution^24^epistatic interaction plays an important role in controlling the expression of complex traits^25^. Post-GWAS analysis of epistatic interactions revealed 11 pairs of epistatic QTL associated with γT and δT contents (Fig. 3). The identification of QTL and the elucidation of their genetic control (main effects and their epistatic effects) are essential for the development of efficient marker-assisted selection (MAS) strategies, aimed at improving breeding efficiency^26^. Haplotype-based association analysis should also be more robust than single-marker analysis because the former utilizes information about LD from multiple markers^27^. In this study, haplotype analysis of QTL identifies nine SNPs that haplotypes at the *qDelt2.1* and *qGam2.1* QTL and identified two haplotypes that appear to have greater effects than the other haplotype groups at these QTL. Similarly, the combination of favorable alleles identified a haplotype, which showed significantly higher γT levels at the *qGam6.1* QTL. These identified haplotypes can be further pyramided in breeding lines using marker-assisted breeding in rice.

In this study, 68 candidate genes were found located within the confidence interval of the identified QTL. Among the genes located near the 18 QTL, the tocopherol *O*-methyltransferase (*OsVTE4*, also known as *γ-TMT*) gene on rice chromosome 2 was of particular interest. This gene is known to be related to vitamin E synthesis, it is involved in the conversion of γT to αT, diminishing the sink of minor tocopherols. Also, *γ-TMT*, located ~ 8 kb away from the *qDelt2.2* QTL, is associated with δT content. Another gene associated with δT content was geranylgeranyl diphosphate synthase (GGDP), located 166 kb from the *qDelt5.1* QTL,

Based on the GWAS results, five lncRNAs were identified. lncRNAs and miRNAs have been identified as key regulatory cascades of eukaryotic transcriptomes^28^. The study of lncRNAs controlling vitamin E biosynthesis in rice is limited. The studies indicate that lncRNAs play important roles in regulating secondary metabolites to improve the self-protection and survival competitiveness of seeds^29^. The interactions between lncRNAs and miRNAs are critical for various biological events using regulating at transcription, post-transcription and epigenetic stages^30^. Thus, exploring these interactions would help us to understand lncRNAs’ functions in greater detail. Cytoscape_v3.9.1 was used to establish the interaction network. Nodes with a higher degree of connectivity to other nodes are often more important in such networks. These nodes are considered hub genetic factors, functional or regulatory elements, with definitive roles in controlling the trait of interest. We identified five lncRNAs (URS00008F1D21_39947 (*Os07g0604466*), URS00008F2D91_39947 (*Os06g0192676*), URS0000180188_39947 (*Os07g0604900*), URS00008E75A9_39947 (*Os02g0821301*), URS00008FD455-39947 (*Os12g0158500*) that could be considered as hub lncRNAs.

In this study, eight TFs were also identified. Promoter analysis showed that *ZFP15*, *ZFP182*, *WRKY77*, *ERF71* and *WRKY66* have binding sites in the promoter of genes involved in the biosynthesis of tocopherols. Two TFs, belonging to the ERF family, including *ERF71* and *EREBP33*, were among these candidate TFs. The first TF resides at *qGam6.1* ~ 160 kb away from SNP id6003412, and is significantly associated with γT. *EREBP33* is located within the QTL *qGam3*.*1*, 244 kb away from SNP id3016979 and is significantly associated with δT content.

It has been shown that vitamin E content increase due to environmental stresses and hormone signaling^21,31^. The members of ERF family have been reported to be responsive in such signaling cues to both endogenous and exogenous alterations and stimulants^32^which suggests their possible co-involvement in the regulation of vitamin E biosynthesis and content. Similarly, members of the WRKY family of TFs, including *WRKY 77* (*qGam1*.*4*, ~ 4 kb from SNP id1012891, associated with γT) and *WRKY 66* (*qDelt2*.*2*, ~ 187 kb from SNP id2012639, associated with δT), have been reported to participate in growth, development, metabolism, and responses to environmental cues^33,34^which again indicates possible co-involvement of such TFs in regulating vitamin E content. The *ZFP15* and *ZFP182* of C2H2 TF families were the other candidate TFs. Both belong to QTL *qGam3*.*1*, ~ 178 kb away from SNP id3016979, associated significantly with γT. Members of this family in rice have been reported to be involved in response to abiotic stresses^19,35^. *NAC70* (*qGam2*.1 and *qDelt2*.*1*, ~ 189 kb away from SNP id2016014, associated with γT and δT) role needs to be further elucidated. *Os03g0818700* (MYB-like) belongs to *qGam3.1* QTL ~ 252 kb away from SNP id3016979 with significant association to γT was also detected (**Supplementary Table S12**). The MYB family has been implicated in ABA response, a hormone previously corroborated to have a substantial role in controlling vitamin E content^36^and also in interacting with other TFs.

Here, three transporter genes, namely *OsAT1*,* ABCI5*, and *Os06g0198800*, located on chromosomes 1, 2, and 6, were identified to be possibly associated with γT transport. *OsAT1* is an anion transporter localized in the endoplasmic reticulum and Golgi that plays a known role in determining grain size and weight^37^(Table 2). *ABCI5*, from the ABC transporter superfamily, is located in the *qGam1.1* QTL, ~ 210 bp away from id1022369. *Os06g0198800* belongs to the SNARE family and is located in the *qGam6.1* QTL, 123 kb away from id6003412. SNARE proteins are a highly conserved superfamily of proteins that mediate vesicle transport between endosomes and trafficking to the plasma membrane in all eukaryotic cells^38^.

The success of the response of plants to environmental stress depends on the regulatory networks that connect plant stress perception and plant responses to these stresses. In these networks, phosphorylation by protein kinases is a key mechanism for activating/deactivating proteins^39^. We identified a few kinases identified in our GWAS. For example, the role of target of rapamycin (*TOR*) as a developmental regulator is widely recognized in both plants and animals, including promoting the upregulation of genes associated with lipid content, and simultaneously downregulating genes involved in stress response and biomolecule degradation^40^. *TOR* (*Os05g0235300)* was observed to be located in the *qDelt5.1* QTL, ~ 181 kb away from id5004299 and was significantly associated with δT levels. A protein-protein interaction network analysis showed that this gene is co-expressed with *VTE4*. qRT-PCR has been proposed for preliminary verification of candidate genes identified by GWAS^40^ and we validated three tocopherol-related biosynthetic genes, including one signaling gene (*TOR*) and two TF genes (*ERF71* and *NAC70*) that may be responsible for δ and γ-tocopherol variation in our natural rice population. The qRT-PCR results showed we do not observe much biosynthesis of vitamin E in the first stages of growth. We observed a significant difference between genotypes #g95 (low), #g171 and #g255 (high) in the mature stage. The results indicate that these 3 genes are involved in the biosynthesis of vitamin E, and further functional studies are warranted to confirm their roles in these processes.

## Conclusions

In this study, a GWAS was carried out using 179 diverse rice accessions with a focus on identifying regulatory genes (TFs, lncRNA and transposons) and other functional genes involved in the transport and signaling of γT and δT biosynthesis. Three important candidate genes *TOR* (*Os05g0235300*), *ERF71*(*Os06g0194000*) and *NAC70* (*Os02g0822400*) were identified and validated using qRT-PCR analysis. To select these candidate genes, various analyses were performed after GWAS, including promoter analysis, lncRNAs-miRNA-mRNA network, RNA-seq and gene expression level analyses. Promoter analysis was conducted to identify potential regulatory elements and binding sites for transcription factors that could influence the expression of candidate genes. The lncRNAs-miRNA-mRNA network analysis helped in understanding the regulatory relationships between non-coding RNAs and protein-coding genes, revealing potential interactions that regulate tocopherol biosynthesis. Additionally, RNA-seq provided insights into the gene expression profiles of diverse rice accessions, which helped in identifying genes that are differentially expressed in relation to tocopherol content, further validating the relevance of the selected candidate genes. In conclusion, our present study unveiled a rich source of genetic elements, including SNPs and putative candidate genes, associated with minor tocopherol biosynthesis in rice. These findings provide a solid foundation for further research on the functional roles of these genetic elements and their potential utilization in breeding programs to improve rice tocopherol.

## Materials and methods

### Rice germplasm collection and SNP genotyping data

The natural population used in this study consisted of 282 rice genotypes collected worldwide and obtained from the International Rice Research Institute (IRRI), Philippines (**Supplementary Table ****S1**), in addition to three local Iranian cultivars (Sadri, Sang Tarom and Dom Siah Kalat). The seeds were planted in augmented block design with local cultivars randomized between the blocks as check varieties in three replicates. Each plot area was 2 × 2 m^2^ with 25 cm within rows spacing. The seeds were sown in Agricultural Research Station at Shavoor, Ahwaz, Iran (48°27’ E, 31°50’ N- 2021–2022). Superphosphate triple and potash (150:150 kg/ha) were applied at the plowing stage. A total of 179 accessions completed the growing season and were used in future studies. These accessions could be grouped into 5 distinct ecotypes: *Indica* (42), *Japonica* (79), *AUS* (21), *Aromatic* (4) and *Admix* (33). All seeds were harvested at maturity and subsequently stored in dry conditions at 4 °C.

Results from the the rice 44.1 K SNPs array for all the studied rice accessions were downloaded from Gramene portal (http://gramene.org)^18^. SNP loci were further filtered to produce a high-quality set of markers with minimum allele frequencies (MAF) greater than 0.05 in TASSEL v5.0^41^. After excluding low quality and monomorphic loci, 34,323 SNP loci were retained.

### Rice grain Tocopherol content analysis

Based on Hu et al. (1996)^42^rice grain tocopherol variants (γ and δ) were separated and quantified using HPLC (LC pump K-1001, KNAUER, Germany) with a C18 reverse phase column (GALAK, 150 mm length, 5 μm particle sizes, 100 A˚, 330 m^2^.g^−1^). Rice grains (2 g) were ground to a fine powder for 1 min and vortex-mixed in *n*-hexane (3:1 solvent: powder) for 1 min at 22 °C. The mixture was wrapped by aluminum foil to reduce light exposure and shaken in a rotary shaker at 220 rpm at 26 ˚C for 24 h. The next day, the samples were centrifuged at 8000 ×g for 10 min at 4 °C. The supernatant was poured into a fresh tube and incubated at 22 ˚C for solvent evaporation. The clear aliquot was filtered through a 0.45 μm PTFE filter and mixed with acetonitrile at a ratio of 1:1.The liquid chromatography was initiated by injecting 20 µl of rice oil sample that followed by eluting using a buffer containing 100% acetonitrile with a flow rate of 1 ml min^−1^ at column temperature of 40 °C. A fluorescent detector (Shimadzu RF − 10Axl, Kyoto, Japan) was used with excitation at 295 nm and emission at 330 nm. A mixed tocopherol standard solution at a concentration of 100 ppm was used as an external standard to accurately quantify the tocopherol content.This standard solution contained a defined mixture of tocopherol isomers, ensuring precise calibration and reliable measurement of tocopherol levels in the analyzed rice samples^43^. Broad-sense heritability (H^2^b: H^2^b = V_G_/V_P_) and narrow-sense heritability (H^2^n: H^2^n = V_A_/V_P_) of the γT and δT contents were estimated using the heritability package in R (https://cran.r-project.org/web/packages/heritability/). Here, V_G_ denotes the genotypic variance, V_P_ is the phenotypic variance, and V_A_ is the additive genetic variance.

### Linkage disequilibrium estimation

The filtered SNP data was used to calculate linkage disequilibrium (LD) between SNPs using r^2^ in a sliding window of 50 markers using TASSEL v5.0^41^. After filtering by MAF > 0.05^20^ and missing genotypic rate from the total of 36,901 SNP markers, 34,323 SNPs remained. All the remaining SNPs were used estimate LD decay across all 12 rice chromosomes. Graphs depicting the decay of LD with physical distance between SNPs were created by aod, dplyr, stringr packages and visualized using ggplot2 in R. The distance across the chromosome when the r^2^ dropped to half of its maximum value was called the LD decay distance^44^.

### Population structure, kinship analysis and GWAS

Polymorphic SNPs (34,323) were used to estimate population structure (Q) and kinship (K). A principal component analysis (PCA) was used to illustrate population classification and was performed using rMVP in R^45^. The kinship analysis used SNP data markers for 179 genotypes. The input file was prepared using TASSEL v.5.0 and the kinship matrix was obtained using the Popkin package in R. A heatmap of kinship relationships was generated using the ggplots package in R. Kurtosis and skewness tests were employed in R to assess the normality of the frequency distribution of phenotypic data. ANOVAs were performed using an augmented block design with the R agricolae package.

Fixed and Random Model with Circulating Probability Unification (FarmCPU) was employed to perform GWAS using rMVP in R^45^. Manhattan plots with suggested threshold lines were produced using rMVP, considering rice with remarkably low LD decay^46^. The interval of significantly associated SNP ± LD decay distance was identified as a QTL. To avoid QTL redundancy, if overlaps existed between QTL flanking areas, they were combined into one QTL^47^.

### Bioinformatics and gene identification

Using the IRGSP-1.0 (https://rapdb.dna.affrc.go.jp/) reference genome, genes underlying the QTL of γT and δT contents that overlapped with the genomic regions (i.e., their associated SNPs), were identified. The flanking region of each peak SNP marker was chosen based on average LD decay^46^ and checked for map order uncertainty and LD. The online Plantpan3 tool (http://plantpan.itps.ncku.edu.tw/)^48^ was used to check each corresponding promoter and co-expression analysis of the main genes (*VTE1*, *VTE2*, *VTE3*, *VTE4*, *VTE5*, *VTE6*, *HPPD*, *TAT*, and *HGGT*) in tocopherol biosynthesis pathway. LncRNAs were identified based on the PLncDB V2.0) https://www.tobaccodb.org/plncdb/) database to explore their potential regulatory roles in tocopherol biosynthesis. The sequence of each lncRNA was taken from the RNA central (https://rnacentral.org/) database. The effect of each associated SNP was annotated using SnpEff: rice genome annotation information from the RAP database (RAP-DB, https://rapdb.dna.affrc.go.jp/) in the SnpEff analysis. Promoter regions of differentially expressed genes (up to 2500 bp from the start codon) were extracted using the Biostrings package in R.

### Haplotyping and epistatic interaction analysis

The SNP haplotype analysis was performed using geneHapR^49^. In this study, favorable haplogroups were defined as the haplotype that showed the highest average values over the other haplotypes. Epistatic effects of significant QTL were obtained via GWAS (− Log_10_ (*p*) > 4.0 in FarmCPU model) and were analyzed for γT and δT. For QQ plot analyses, the snpPairInteraction function in the FRGEpistasis of R package^50^ was used and determined to be significant at *p*-value ≤ 0.05. The Circlize package was used to visualize the significant epistatic interactions.

### RNA-Seq and quantitative real-time PCR analysis of candidate genes

For candidate genes expression pattern analysis, first, we performed a differential expression pattern analysis at different tissues that were obtained from the Rice Genome Annotation Project database (RGAP, http://rice.plantbiology.msu.edu/), including leaves-20 days (library name in NCBI SRA; SRX100741), seed-5 DAP (SRX100749), embryo-25 DAP (SRX100753), and endosperm- 25 DAP (SRX100754). A heatmap was generated to visualize the gene expression patterns across the different tissues.

Three selected genotypes, #g95 (low tocopherol), #g255 and #g171 (high tocopherol), were grown in spring 2023, and fertilized three times with complete fertilizer. The genotypes were sampled at milky (7 days post pollination (DAP)), doughy (14 DAP) and mature developing seed (28 DAP) stages. Sampled seeds were wrapped in foil and placed in liquid nitrogen. Total RNA was isolated from seeds in different developmental stages using GenUP™ Total RNA Mini Kit (biotechrabbit GmbH, Berlin, Germany). First-strand cDNA was synthesized from total RNA using Thermo Scientific RevertAid RT Kit (#K1691, Thermo Fisher Scientific). qRT-PCR was performed using SYBR Green (FP205, Tiangen) reagents on Real-Time PCR machine (T100 Thermal Cycler, Bio-Rad). All qRT-PCRs were performed in three independent replicates and the relative expression levels were calculated using 2^–∆∆Ct^ method^51^. The *OsActin* (*Os03g0718100*) was used as an internal control for expression normalization. Primer pairs used for the rice cDNA amplification were designed using Primer3Plus (https://www.primer3plus.com**Supplementary Table S11**).

### Construction of miRNA-lncRNA-mRNA network

To identify lncRNAs as miRNA targets in vitamin E biosynthesis pathway, the lncRNAs were subjected to BLAST (http://blast.ncbi.nlm.nih.gov/Blast.cgi)^52^ against identified miRNAs registered for rice in miRBase^53^. The miRNA targets as mRNA were identified by psRNATarget (http://plantgrn.noble.org/psRNATarget/) with expectation ≤ 3 by giving miRNA sequences as input and searching against the mRNA sequences of 13 main genes^21^ and 20 transcription factors (data not shown) involved in vitamin E biosynthesis. The mRNA targets for all the lncRNAs were identified using lncTAR (http://www.cuilab.cn/lnctar)^54^. A high normalized deltaG (nDG) threshold (−0.20) was set to obtain high confidence lncRNA-mRNA interacting pair. The Cytoscape v3.9.1 software was used for network mapping of lncRNAs and related miRNAs and mRNAs.

### Gene ontology (GO) and functional enrichment analysis

ShinyGO v0.75 (http://bioinformatics.sdstate.edu/go/)^55^ was used for GO analysis. The top 10 GO terms from each category (BP, CC, and MF) were extracted and incorporated in various enrichment plots (SRplot and GO: http://bioinformatics.com.cn/en). Functional enrichment analyses of candidate genes were depicted using MapMan (ver. 3.6.0RC1) software based on the functional annotated file osa_RAPDB_mapping (https://MapMan.gabipd.org/MapManstore).

## Supplementary Information

Below is the link to the electronic supplementary material.

Supplementary Material 1

Supplementary Material 2

## Data Availability

All relevant data can be found within the manuscript and its supporting materials and further inquiries can be directed to the corresponding author/s.

## Abbreviations

TF: transcription factor
QTL: quantitative trait loci
LD: linkage disequilibrium
SNP: single nucleotide polymorphism
DMGGBQ: dimethylgeranylgeranylbenzoquinol
DMPBQ: dimethylphytylbenzoquinol
GGDR: geranylgeranyl diphosphate reductase
GGPS: geranylgeranyl diphosphate synthase
HPPD: hydroxyphenylpyruvate dioxygenase
HPT: homogentisate phytyl transferase
MPBQ-MT: MPBQ methyltransferase
TAT: tyrosine aminotransferase
TC: tocopherol cyclase
TMT: tocopherol methyltransferase
ABH: alpha/beta hydrolase
PK: Phytol kinase
PPK: Phytyl phosphate kinase
GWAS: Genome-Wide Association Studies
DEGs: Differentially expressed genes
GO: Gene Ontology
δT: Delta-tocopherol
γT: Gamma-tocopherol
