# Supplementary material for: Genome-wide association mapping and transcriptional analysis uncover genetic determinants of minor tocopherols in rice seeds
Source: Sci Rep. 2025 Aug 5;15:28530. doi: 10.1038/s41598-025-14473-3 (PMC12325677; doi:10.1038/s41598-025-14473-3)
Supplement: Supplementary file 1 — Supplementary Material 1 [file 41598_2025_14473_MOESM1_ESM.docx]

**
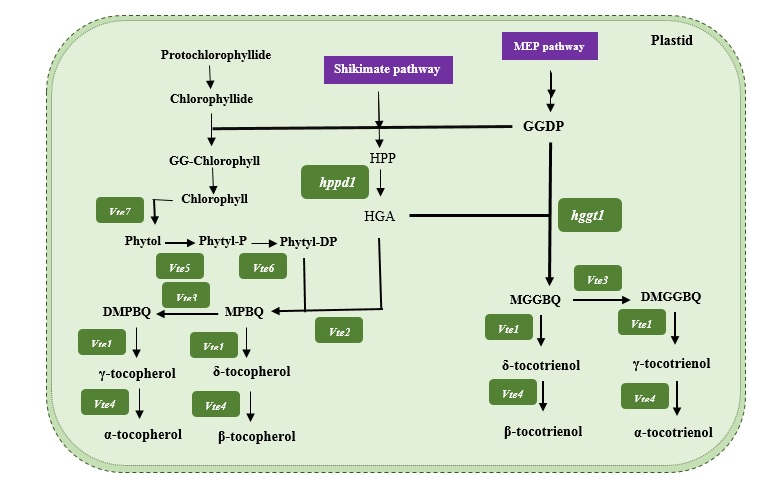
**

**Supplementary Fig. S1. Simplified model of the tocopherol biosynthesis pathway.** The shikimate pathway provides HGA as the precursor for tocopherol synthesis. PDP as the lipid precursor is derived from free phytol, released during chlorophyll degradation. Prenylation of HGA with PDP resulting in MPBQ production is the key step for tocopherol biosynthesis. Methylation of MPBQ yields DMPBQ. *Vte1* converts MPBQ and DMPBQ to δT and γT, respectively. Vte4 methylates δT and γT to βT and αT, respectively (created at BioRender.com.).


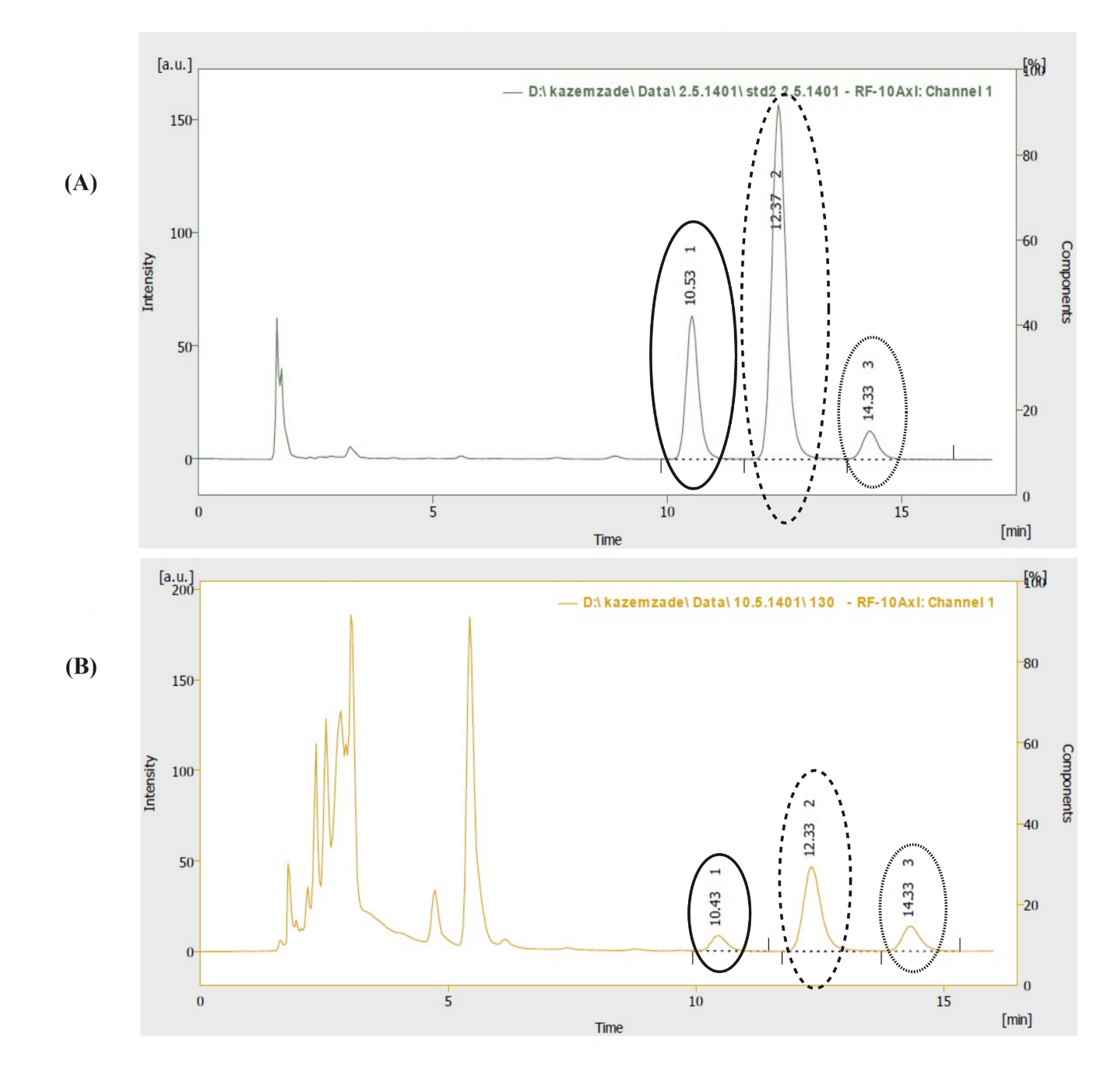


**Supplementary Fig. S2. Chromatograms of the vitamin E isomers in rice**. **)A)** Mixed standard samples of three tocopherol isomers, **)B)** Rice sample. Solid line: δ, dashed line: γ, point: α isoform.


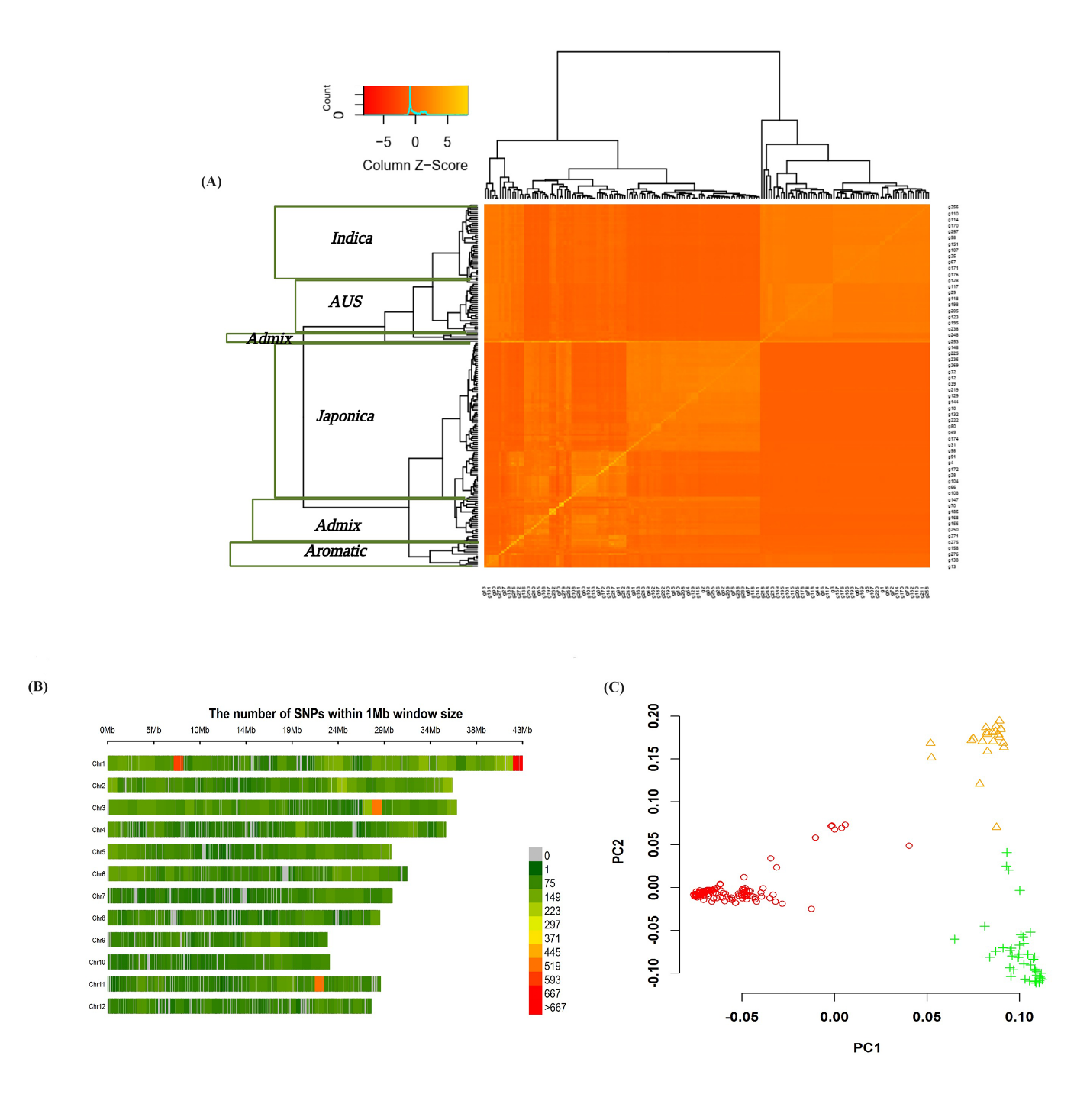


**Supplementary Fig. S3. (A)** Van Raden kinship matrix - heat map of the marker-based kinship (K) matrix for 179 rice accessions. Matrix obtained from 34,323 SNPs. **(B)** The chromosomal distribution of the used SNPs within 1 Mbp window size. The number of markers on each chromosome varies, with chromosome 9 having the least markers and chromosome 1 having the highest. **)C)** PCA plot used to analyze the population structure showing three major subpopulations (shown in different colors) in the current panel.


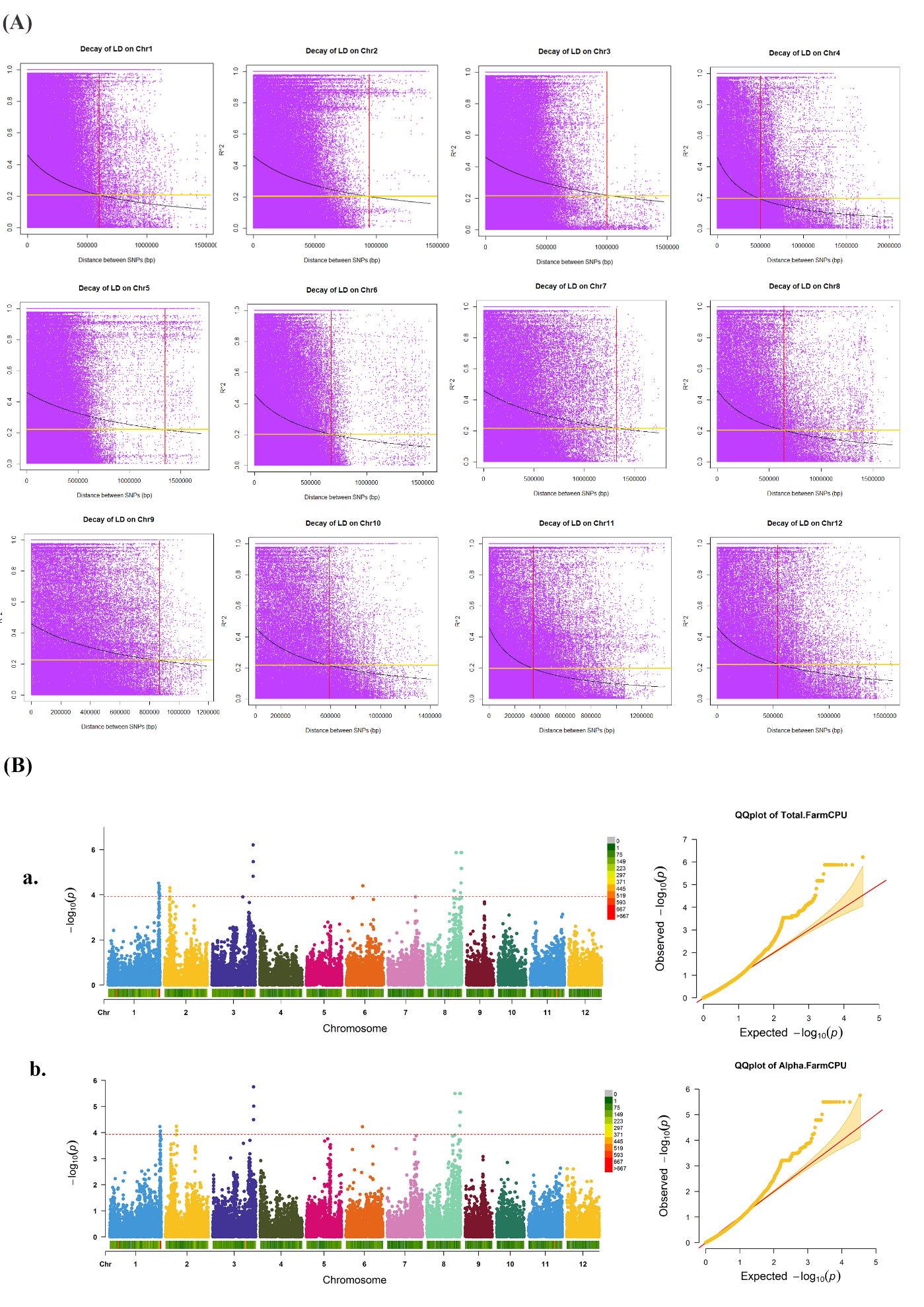


**Supplementary Fig. S4.** The decay of LD along physical distances on 12 rice chromosomes was computed using SNP data of 179 rice accessions. A scatter *r*^2^ against physical distance showed a clean pattern of LD decay in the 179 rice accessions. A critical value of the determination coefficients *r*^2^ > 0.2 was determined to be the threshold for LD decay.

**
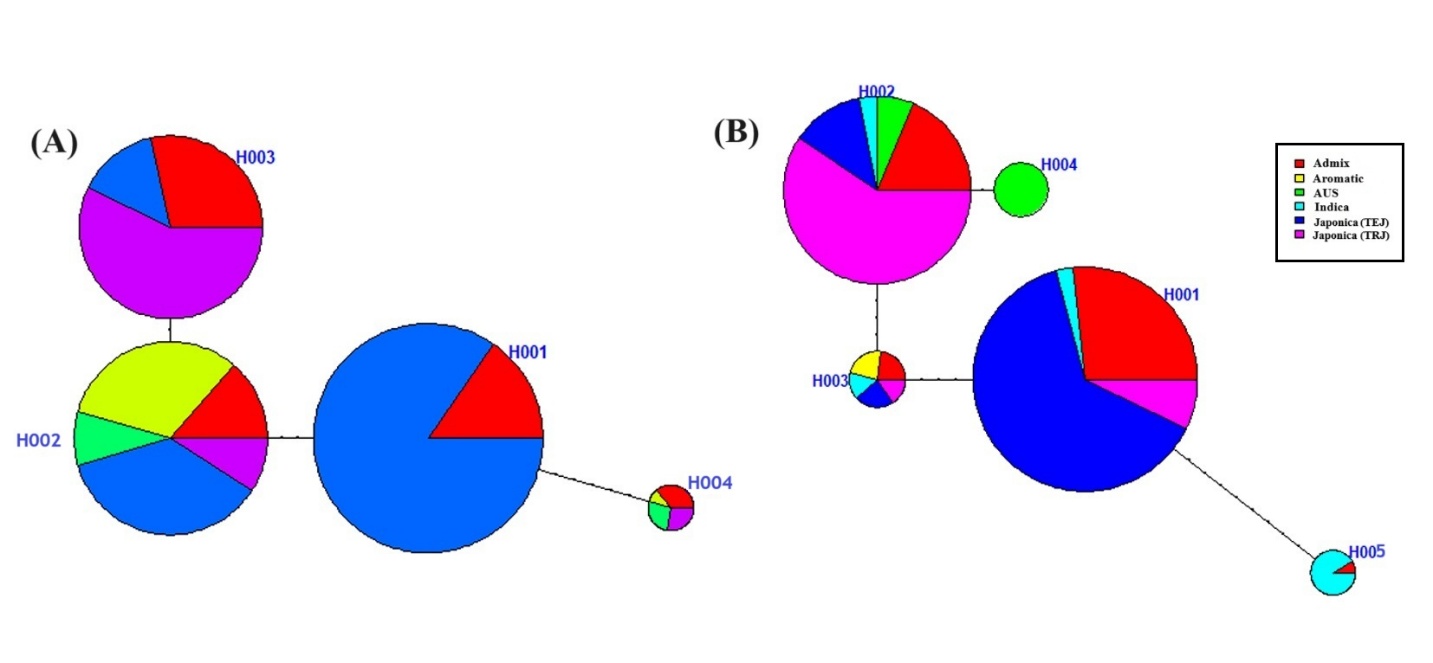
**

**Supplementary Fig. S5.** Haplotype network of **)A)** *Delt2.1, qGam2.1* **)B)** *qGam6.1* Each circle represents a haplotype and the size indicates the accession number. The pies in different colors represent the ratio of category in each haplotype.
